# The C_2_H_2_ transcription factor SltA is required for germination and hyphal development in *Aspergillus fumigatus*

**DOI:** 10.1128/msphere.00076-23

**Published:** 2023-06-01

**Authors:** Tim J.H. Baltussen, Norman van Rhijn, Jordy P.M. Coolen, Jan Dijksterhuis, Paul E. Verweij, Michael J. Bromley, Willem J.G. Melchers

**Affiliations:** 1 Department of Medical Microbiology, Radboud Institute for Molecular Life Sciences, Radboud University Medical Center, Nijmegen, The Netherlands; 2 Manchester Fungal Infection Group, Division of Evolution, Infection and Genomics, Faculty of Biology, Medicine and Health, University of Manchester, Manchester, UK; 3 Westerdijk Fungal Biodiversity Institute, Utrecht, The Netherlands; University of Georgia, Athens, Georgia, USA

**Keywords:** *Aspergillus fumigatus*, germination, SltA, hyphal development, transcriptional regulation

## Abstract

Germination of inhaled *Aspergillus fumigatus* conidia is a necessary sequitur for infection. Germination of conidia starts with the breaking of dormancy, which is initiated by an increase of the cellular perimeter in a process termed isotropic growth. This swelling phase is followed by polarized growth, resulting in the formation of a germ tube. The multinucleate tubular cells exhibit tip growth from the hyphae, after which lateral branches emerge to form the mycelial network. The regulatory mechanisms governing conidial germination are not well defined. In this study, we identified a novel role for the transcription factor SltA in the orchestration of germination and hyphal development. Conidia lacking *sltA* fail to appropriately regulate isotropic growth and begin to swell earlier and subsequently switch to polarized growth faster. Additionally, hyphal development is distorted in a ∆*sltA* isolate as hyphae are hyper-branching and wider, and show branching at the apical tip. ∆*sltA* conidia are more tolerant to cell wall stressors on minimal medium compared to the wild-type (WT) strain. A transcriptome analysis of different stages of early growth was carried out to assess the regulatory role of SltA. Null mutants generated for three of the most dysregulated genes showed rapid germ tube emergence. Distinct from the phenotype observed for ∆*sltA*, conidia from these strains lacked defects in isotropic growth, but switched to polarized growth faster. Here, we characterize and describe several genes in the regulon of SltA, highlighting the complex nature of germination.

**IMPORTANCE:**

*Aspergillus fumigatus* is the main human fungal pathogen causing aspergillosis. For this fungus, azoles are the most commonly used antifungal drugs for treatment of aspergillosis. However, the prevalence of azole resistance is alarmingly increasing and linked with elevated mortality. Germination of conidia is crucial within its asexual life cycle and plays a critical role during the infection in the human host. Precluding germination could be a promising strategy considering the role of germination in *Aspergillus* spp. pathogenicity. Here, we identify a novel role for SltA in appropriate maintenance of dormancy, germination, and hyphal development. Three genes in the regulon of SltA were also essential for appropriate germination of conidia. With an expanding knowledge of germination and its different morphotypes, more advances can be made toward potential anti-germination targets for therapy.

## INTRODUCTION

The *Aspergillus* species have a worldwide distribution and grow saprotrophically on a wide variety of dead organic matter but can also act as endophytes (1, 2). They appear in a number of (pathogenic) interactions as on coral (*A. sydowii*) (3) and grain and corn (*A. flavus*) (4), and as post-harvest pathogens (e.g., *A. niger* on onions and hyacinth bulbs) (5). *Aspergillus* species-induced human diseases include allergic bronchopulmonary aspergillosis, chronic pulmonary aspergillosis, invasive pulmonary aspergillosis, severe asthma with fungal sensitization, and extrapulmonary aspergillosis (6). The majority of *Aspergillus* infections in humans can be attributed to *A. fumigatus* (7).

The filamentous fungus *A. fumigatus* produces multinucleate tubular cells termed hyphae. Hyphal tip extension occurs through the synthesis and addition of a new cell wall and membrane via vesicles fusing with the apical plasma membrane (8). This highly polar extension of the tip helps *A. fumigatus* to penetrate and invade blood vessels and tissue, which is a characteristic of invasive aspergillosis. Before the fungus grows in a polarized manner, the conidium breaks dormancy and the reactivated cell expands isotropically (9, 10). This swelling phase is characterized by intracellular trehalose and mannitol degradation, water uptake, and a decrease in the microviscosity of the cytoplasm (11, 12). The swollen conidium undergoes switching from isotropic to polarized growth, which is characterized by localized vesicle fusion and membrane extension leading to a tubular outgrowth known as the germ tube. The germ tube extends from the tip and subsequently branches subapically to form a network of hyphae. During the extension of the hyphal tip, the Spitzenkörper (Spk) is important for polarity maintenance. The Spk is a dense cluster of vesicles observed at the growing tip area close to the apical plasma membrane, together with several protein complexes and cytoskeleton components, such as microtubules and actin filaments (8, 13).

Germination of *Aspergillus* spp. is highly dependent on nutrient availability such as a carbon source, inorganic phosphate, inorganic nitrogen, and magnesium sulfate (9, 14, 15). Despite the importance of germination in the initiation of *Aspergillus* disease, the genetic factors that govern this process remain largely unresolved. In this study, we identify that the zinc-finger C_2_H_2_ type transcription factor (TF) SltA is functionally linked to the control of germination as it has a non-redundant role in controlling emergence from dormancy, germ tube formation and temporal and spacial control of hyphal branching. The ∆*sltA* strain was recently studied for virulence and showed a reduced capacity to adhere to, invade, and damage pulmonary epithelial cells (16, 17). Additionally, ∆*sltA* showed increased susceptibility to cell membrane stressors (16). In the current study, however, the focus was on germination and germ tube elongation. Furthermore, we show that genes under regulatory control of SltA are required for appropriate maintenance of dormancy and germination.

## MATERIALS AND METHODS

### Fungal strains

The MFIG001 strain (WT), a member of the CEA10 laboratory lineage deficient in nonhomologous end joining (18), was used as the parental isolate to generate the TF knockout library (19). Briefly, gene replacement cassettes were generated using a fusion PCR approach. The hygromycin B phosphatase cassette (*hph*) was amplified from pAN7.1 and around 1 kb of the 5′ and 3′ flanks from each target gene were amplified. The fusion PCR cassette was used to transform protoplasts as previously described (20). The strains used in this study are given in Table 1.

**TABLE 1:** *Aspergillus fumigatus* strains used in this study^*a*^ ^,^ ^*b*^

Strain	Parent	Genotype	Name	Source or reference
A1160+	CEA10	∆*ku80::pyrG*	MFIG001	MFIG
2C7	A1160+	∆*sltA::hph*	∆*sltA*	MFIG
2G1	A1160+	∆*AFUB_051540::hph*		MFIG
2G12	A1160+	∆*rfeG::hph*	∆rfeG	MFIG
2G7	A1160+	∆*steA::hph*	∆*steA*	MFIG
2H2	A1160+	∆*AFUB_056790::hph*		MFIG
3B2	A1160+	∆*seb1::hph*	∆*seb1*	MFIG
3B6	A1160+	∆*amyR::hph*	∆*amyR*	MFIG
3B7	A1160+	∆*rfeF::hph*	∆*rfeF*	MFIG
5G5	A1160+	∆*AFUB_068500::hph*		MFIG
5H1	A1160+	∆*zfpA::hph*	∆*zfpA*	MFIG
6A2	A1160+	∆*AFUB_016450::hph*		MFIG
7B8	A1160+	∆*medA::hph*	∆*medA*	MFIG
7E8	A1160+	∆*AFUB_073740::hph*		MFIG
2C7rec	2C7	*sltA::ptrA*	sltArec	This study
41A2	A1160+	∆*AFUB_035430::hph*		MFIG
74B6	A1160+	∆*AFUB_071880::hph*		MFIG
86E11	A1160+	∆*AFUB_085360::hph*		MFIG
85F9	A1160+	∆*AFUB_084520::hph*		MFIG
99H5	A1160+	∆*AFUB_099730::hph*		MFIG

^*a*^ hph, hygromycin B phosphatase cassette.

^*b*^ MFIG, Manchester Fungal Infection Group.

### Construction of complementation strain

A knock-in cassette was generated using a fusion PCR approach to complement the deleted gene for ∆*sltA* (AFUB_041100). Primers 041100recP1 and 041100recP2 were used to amplify the AFUB_041100 gene with 50 bp upstream flanking region and the terminator (500 bp), while primers 041100recP3 and 041100recP4 were used to amplify the downstream flanking region (500 bp) of the AFUB_041100 gene from MFIG001 genomic DNA. Primers ptrA_F_linker1 and ptrA_R_linker2 were used to amplify a 2 kb pyrithiamine resistance (*ptrA*) cassette from plasmid pSK485. The three products were fused together with the nested primers 041100recP5 and 041100recP6 and the common linker sequences on primers 041100recP2 and ptrA_F_linker1, and 041100recP3 and ptrA_R_linker2 using the fusion PCR protocol previously described (21).

Our transformation was based upon a clustered regularly interspaced short palindromic repeat Cas9 protocol from van Rhijn et al., which used a ribonucleoprotein (RNP) assembly method previously described by Al Abdallah et al. (22, 23). Briefly, target-specific crRNAs were designed using the web-based tool EuPaGDT. The genome sequence of *A. fumigatus* A1163 was manually uploaded to EuPaGDT, and the program was carried out using default settings to design to the AFUB_041100 locus. The crRNA closest to the target integration site with the highest QC score was manually selected for transformation. The RNP complexes were assembled *in vitro* by mixing Cas9 V3 protein, a 67-mer tracrRNA and locus-specific crRNA (Integrated DNA Technologies). The knock-in cassette and RNP complexes were mixed with purified protoplasts, and transformation was carried out in the previously knocked out strain (∆*sltA*) as described by van Rhijn et al. (22).

Validation of the homologous recombination was performed by PCR using primers 041100P5 and 041100recP2, and 041100recP3 and 041100P4. The sequences of all primers and crRNAs used are given in Table S7.

### Microscopy

Mutant strains and parental WT strain were grown on Sabouraud dextrose agar (SDA; dextrose 40 g/L, peptone 10 g/L) slants for 3 d at 37°C, followed by room temperature for 3–7 d. Conidia were harvested with a 0.05% (vol/vol) Tween 20 aqueous solution and filtered through a 40 µm nylon cell strainer (BD Falcon). To analyze the germination of the mutant and WT strains, the appropriate amount of conidia was inoculated into liquid RPMI-1640 medium (Gibco Life Technologies) supplemented with 2% glucose, buffered with MOPS (3-(*N*-morpholino)propanesulfonic acid) to a final concentration of 5 × 10^5^ spores/mL in a 96-well plate, and incubated for 0 h, 2 h, 4 h, 6 h, 8 h, 10 h, 12 h, and 16 h at 37°C. Conidia were fixed in 4% formaldehyde, stained with Blankophor (1 mM stock, diluted to 250 µM in RPMI-1640), and mounted on a microscope slide for viewing. Blankophor staining was imaged using a Zeiss Axio Scope A1 microscope with an objective EC “Plan-Neofluar” 40x/0.75 M27 lens using 330–385 nm excitation and 420 nm emission. Images were captured with a Zeiss Axiocam 208 color using Zen 3.3 blue edition software. Images were used to calculate the germination rate after 6 h and 8 h of development for the WT and ∆*sltA* strains. For this, three experiments using three replicates were performed, and a minimum of 100 conidia were scored. Conidia having an emerging germ tube (teardrop shape) were scored as germinated. *P*-values were calculated using the Mann-Whitney-Wilcoxon Test. Data were tested for equality of variance and normality using the Bartlett’s test and Shapiro-Wilkinson’s test, respectively (24, 25).

For the cryo-scanning electron microscopy (cryo-SEM), WT and ∆*sltA* conidia were counted using a hemacytometer, and 10,000 conidia in 1 µL were inoculated onto solid RPMI-1640 plates and incubated for 16 h at 37°C. Agar containing the (germinating) conidia was selected under a binocular, excised with a surgical blade (no. 11, Swann Morton Limited, Sheffield, UK) as small agar (approx 3 × 3 mm) blocks, and transferred to a copper cup for snap-freezing in nitrogen slush. Agar blocks were glued to the copper surface with silicon crease (Walker-Chemie, Munich, Germany). Samples were examined in a JSM-IT200LV scanning electron microscope (JEOL, Tokyo, Japan) equipped with an Oxford CT1500 Cryostation for cryo-SEM. Electron micrographs were acquired from uncoated frozen samples, or after sputter-coating by means of a gold target for 2–3 times for 1 min. Micrographs of uncoated samples were taken at an acceleration voltage of 2 kV and at 5 kV in case of the coated samples.

For the time-lapse live cell imaging, 5 × 10^4^ conidia of WT, ∆*sltA*, and sltArec were inoculated in a 24 well glass bottom plate (Greiner Bio-One) in RPMI-1640. Growth was assessed up to 16 h at 37°C by taking an image every 20 min on a Leica SP8 confocal microscope using a 10x/0.4 NA lens and a 514 nm argon laser. Images were compiled into videos using ImageJ.

### Phenotypic analysis of germination and hyphal growth

Swelling of conidia and germ tube formation was monitored for 16 h at 37°C using an oCelloScope and UniExplorer software (version 11.1.0.8756) (BioSense Solutions Aps, Denmark). For each strain, 5 × 10^3^ conidia were inoculated per well in quadruplicate in RPMI-1640, and the 96-well plates were centrifuged for 10 min at 2,500 rpm (Rotanta 460R, Hettich Zentrifugen) to collect them at the bottom of the wells. Objects were scanned every hour for the first 8 h and every 30 min during the next 8 h. Using the UniExplorer software, the area of the conidia was measured together with the circularity of the cells. To select only single conidia, the data were filtered by setting the area threshold between >10 μm^2^ and <25 μm^2^ and circularity >1.00. Additionally, non-growing cells were removed by setting the area threshold after 8 h of growth to >25 μm^2^. In total, between 52 and 107 cells were tracked for each analyzed strain over a growth period of 16 h. Area and circularity after each hour of growth was compared between the strains and *P*-values were calculated by Kruskal-Wallis’s test and Dunn’s correction (26 - 28). Data were tested for equality of variance and normality using the Bartlett’s test and Shapiro-Wilkinson’s test, respectively (24, 25). Germination rate was calculated by using the circularity measure, which drops when germ tubes start to emerge and was set to <0.98. The DescTools package was used to calculate the 95% confidence interval (CI). The BinomCI function was used to provide CIs for two levels of a nominal variable using the Wilson method (29). To test whether the germination rates were significantly different between the strains, the function prop.test() was used as binomial test in R. For each strain, the mean area in μm^2^ and the SD of the mean were calculated for dormant conidia and after 4 h of growth. Using the area increase in μm^2^, the mean percentage of conidial swelling was calculated.

Video microscopy files were analyzed using ImageJ 1.52o software (30). For the number of branch events, we measured 50 events for each strain (i.e., WT, ∆*sltA*, and sltArec) after 12 h and 16 h of growth. For the analysis of the tip splitting phenotype, the distance from newly emerging branch to the apical tip was measured for 75 events during 16 h of growth for each strain. For analysis of the hyphal elongation rate, the length of 20 elongating hyphae was measured between 14 h and 16 h of growth to calculate the elongation speed. For analysis of the hyphal diameter, the width of 50 hyphae was measured at a random location but at least at 20 µm distance from the hyphal tip. *P*-values were calculated by Kruskal-Wallis’s test and Dunn’s test with Benjamini-Hochberg correction (26 - 28). Data were tested for equality of variance and normality using the Bartlett’s test and Shapiro-Wilkinson’s test, respectively (24, 25).

### Radial growth and cell wall stress experiments

Radial growth of the WT, ∆*sltA*, and sltArec strain was measured by spotting 100 spores in 4 µL on minimal medium (MM) containing per liter: 10 g glucose, 5.95 g NaNO_3_, 0.522 g KCl, 1.5 g KH_2_PO_4_, 50 mg MgSO_4_·7H_2_O, 1 mL trace elements. Trace elements contained (per 200 mL): 10 g EDTA, 4.4 g ZnSO_4_·7H_2_O, 1.01 g MnCl_2_·4H_2_O, 0.315 g CuSO_4_·5H_2_O, 0.22 g (NH_4_)_6_Mo_7_O_24_·4H_2_O, 1.0 g Fe(II)SO_4_·7H_2_O, and 2.2 g H_3_BO_3_ (31). Plates were incubated at 37°C for 72 h, and colony radius was measured every 24 h.

Stock solutions of caspofungin (caspo), Congo red (CR), and calcofluor white (CFW) were prepared in dimethyl sulfoxide (DMSO) to a concentration of 3,200 mg/L, 2,000 mg/L, and 2,000 mg/L, respectively. The 96-well plates were prepared with twofold dilutions in RPMI-1640 medium supplemented with 2% glucose and buffered with MOPS. The caspo, CR, and CFW concentrations ranged from 0.016 to 8 mg/L, 2 to 1,000 mg/L, and 2 to 1,000 mg/L, respectively. Conidia were harvested with a 0.05% (vol/vol) Tween 20 aqueous solution, and the suspension was adjusted to 80–82% transmission at 530 nm using a spectrophotometer (Genesys 30, Thermo Fisher Scientific) to create a suspension of 1–4.2 × 10^6^ spores/mL. Spores were inoculated into the 96-well plates to create a final concentration of 2–5 × 10^5^ spores/mL. The plates were incubated for 48 h at 37°C to visually check growth and determine appropriate concentration for the stress experiments.

To study the effect of cell wall antifungals on the growth of the WT, ∆*sltA*, and sltArec strain, radial growth was measured as described previously on MM plates supplemented with 0.5 mg/L caspo, 31 mg/L CR, or 31 mg/L CFW in triplicate. Additionally, serial 10-fold dilutions of conidia ranging from 1 × 10^4^ to 1 × 10^2^ cells in a volume of 5 µL were spotted onto MM supplemented with 0.5 mg/L CF, 31 mg/L CR, or 31 mg/L CFW in triplicate. Plates were incubated at 37°C for 48 h and imaged to determine the relative sizes of the colony.

### Transcriptomics using RNA sequencing

WT and ∆*sltA* strains were grown on SDA slants for 4 d at 37°C. For RNA extractions, 1 × 10^5^ spores/mL of both strains were grown in 50 mL of RMPI-1640 medium supplemented with 2% glucose and buffered with MOPS for 0 h, 2 h, 4 h, 6 h, 8 h, 12 h, and 16 h at 37°C with constant shaking at 150 rpm in triplicate. Liquid cultures were centrifuged for 5 min at 2,000 rpm (Rotanta 460R, Hettich Zentrifugen), RPMI-1640 medium was removed, and pellets were transferred to 1.5 mL tubes containing glass beads, immediately frozen with liquid nitrogen and kept at –70°C until use. Frozen samples were homogenized with a MagNa lyser (Roche) by three times shaking for 30 s at 7,000 speed. One milliliter of TRIzol reagent (Ambion Life Technologies) was added, immediately vortexed for 1 min and incubated at room temperature for 5 min. Two hundred microliters of chloroform was added, vigorously shaken for 15 s, incubated at room temperature for 3 min, and centrifuged at 12,000× G for 15 min at 4 °C (Hettich^®^ MIKRO 220R). Clear supernatant was transferred to a new 1.5 mL centrifuge tube, 200 µL of chloroform was added, vigorously shaken for 15 s, incubated at room temperature for 3 min and centrifuged at 12,000× G for 15 min at 4°C. Clear supernatant was transferred to a new 1.5 mL centrifuge tube, 500 µL of isopropanol was added, and samples were mixed by inversion and incubated for 2 h at –20°C. Next, samples were centrifuged at 12,000× G for 10 min at 4°C, supernatant was removed and pellets were washed with 70% ethanol; 0 h and 2 h pallets were resuspended in 30 µL Diethyl pyrocarbonate (DEPC)-treated H_2_O, and 4–16 h pallets were resuspended in 50 µL DEPC-treated H_2_O. The concentration of RNA was measured with a Qubit 4 fluorometer (Invitrogen, Thermo Fisher Scientific) using RNA High Sensitivity Assay kit, and the quality was assayed with a TapeStation 4150 system (Agilent) using a High Sensitivity Screentape.

The mRNA library was constructed using Illumina Stranded mRNA prep protocol according to manufacturer’s instruction (Illumina Inc.). Briefly, RNA samples were purified, fragmented, and used to synthesize cDNA. An adenine (A) nucleotide was added to the 3ʹ ends of the blunted fragments. Next, preindex anchors were ligated to the double-stranded cDNA fragments. Finally, the adapter-ligated fragments were purified using magnetic beads and amplified to add indexes and primer sequences for cluster generation. Sequencing was performed on a NovaSeq6000 with a 2 × 150 bp S1 flow cell (Illumina Inc.).

### Transcriptomic analysis

Illumina reads were cleaned of adapter sequences and mapped to the reference genome (*A. fumigatus* A1163 from Ensembl release 52) using STAR version 2.7.3a (32). Counting number of reads per gene was done using quantMode GeneCounts with alignIntronMax 1000. Data analyses were carried out using the R programming language (version 4.1.1) (33). Count data were used to generate a DESeqDataSet using the DESeq2 package, version 1.34.0 (34), and genes with no to very few reads were removed (i.e., genes with row sum of ≤10). The regularized log transformed data were used for exploratory analysis and data visualizations. Principal component analysis (PCA) plots were generated using the packages ggplot2 version 3.3.5 and PCAtools version 2.6.0 (35, 36). Samples were grouped per strain (i.e., ∆*sltA* and WT) and growth phase (i.e., dormant (0 h samples), isotropic growth (2 h and 4 h samples), polarized growth (6 h and 8 h samples), and early hyphal growth (EHG) (12 h and 16 h samples) to compare transcript levels of ∆*sltA* to WT in each growth phase. The normalization and dispersion estimations were performed with DESeq2 using the default settings. Differentially expressed genes (DEGs) were identified by filtering the results for each growth phase using the following thresholds: log_2_ fold change (Log2FC) ≥1 or ≤–1; adjusted *P*-value ≤0.05 (Benjamini-Hochberg) (28).

### Gene set and secondary metabolite cluster enrichment analysis

Functional category (FunCat) enrichment analyses were carried out with the *A. fumigatus* A1163 annotation using the FungiFun2 2.2.8 Beta web-based tool (37). DEGs showing a Log2FC ≥1 or ≤–1 and adjusted *P*-value ≤0.05 were grouped together per growth phase and subjected to the enrichment analysis. Significance levels of the enrichment were analyzed using the default settings (i.e., *P*-value of <0.05 with a Benjamini-Hochberg adjustment (28)), except for the background genes; as background, the 9,947 input genes of the expression analysis were used. Secondary metabolites clusters were obtained from Bignell et al. (38). Manual curation was performed to find genes present in A1163.

## RESULTS

### Screening of TFs expressed during germination and characterization of ∆*sltA* germination and hyphal morphogenesis phenotype

**Fig 1:**
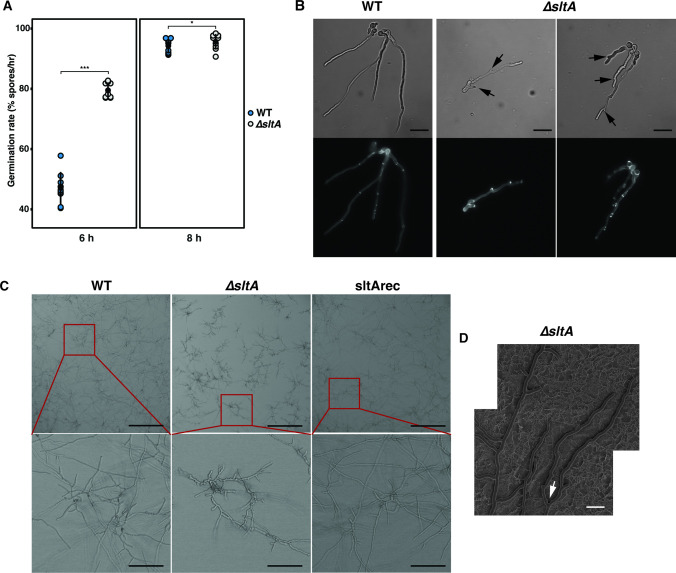
Hyphal morphology of WT, ∆*sltA*, and sltArec strains. (**A**) Germination rate after 6 h and 8 h of the WT and ∆*sltA* strains. *P*-values were calculated using the Mann-Whitney-Wilcoxon Test. *, *P*-value <0.05; ***, *P*-value <0.001. (**B**) Bright-field and fluorescent microscopy images of WT and ∆*sltA* strains after 16 h of growth in RPMI-1640 at 400× magnification. Black arrows indicate the blunted hyphal tips, size bars represent 10 µm. (**C**) WT, ∆*sltA,* and sltArec hyphal morphology after 16 h of growth in RPMI-1640, size bars represent 400 and 85 µm in the higher magnification panels. ∆*sltA* showed abnormal hyphal formation and apparent polarity defect with hyper-branching and blunted tips. (**D**) Cryo-scanning electron microscopy (cryo-SEM) of growing ∆*sltA* hyphae after 16 h on solid RPMI-1640 agar. White arrow indicates splitting of the apical tip, size bar represents 20 µm) Two cryo-SEM pictures were merged together to show a larger area of the hyphae (original pictures in see Supplementary files 3 at https://figshare.com/s/f7986b59ca3cf3ef4abf).

We have previously shown that 13 TFs have enhanced expression (i.e., ≥ fourfold) when the transcriptomes of *A. fumigatus* at the isotropic growth phase were compared with those from dormant spores or when the polarized growth phase was compared with the isotropic growth phase (Table S1) (39). To ascertain if the 13 TFs had a role in regulating these processes, we examined rates of conidial germination and hyphal tip growth of corresponding TF null mutants (19) by bright-field and fluorescence microscopy. Early germ tube emergence (Fig. 1A) and a markedly unstable hyphal morphogenesis in ∆*sltA* were observed such as blunted tips (indicated by the arrows in Fig. 1B) and meandering hyphae, which were not apparent in the WT and other TF null mutants used in this study. Fig. 1B shows the WT hyphal phenotype (i.e., straight elongating hyphal tips) and the unstable ∆*sltA* hyphal morphogenesis (i.e., defect in hyphal tip branching, blunted tips and meandering hyphae). Our exploratory microscopic data suggest that SltA is involved in regulating genes involved in germination and hyphal growth. Consequently, the ∆*sltA* strain was selected for more in-depth analysis of germination and EHG considering its abnormal morphological phenotype.

**Fig 2:**
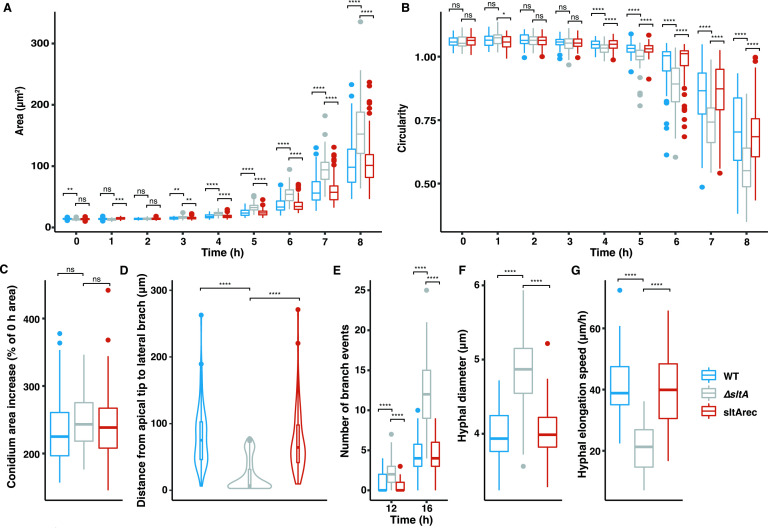
SltA is required for maintenance of dormancy, appropriate germination, and hyphal development. (**A**) Fungal area of the WT, ∆*sltA*, and sltArec strains was measured during 8 h of germination at 37°C. (**B**) Fungal object circularity of the WT, ∆*sltA*, and sltArec strains was measured during 8 h of germination. Round conidia have a circularity of >1.00; this number decreases when cells switch to polarized growth. (**C**) Conidium area increased in percentage compared with the 0 h area, i.e., conidial area before germ tube emergence was compared with the conidial area at 0 h. (**D**) Distance from the tip of the apical hyphae to the location where a new lateral branch emerged. (**E**) Number of branch events per conidium after 12 h and 16 h of growth for each strain. (***F***) Hyphal diameter of each strain measured at a random location but at least 20 µm from the growing tip. (**G**) Hyphal elongation speed in μm per hour for each strain. *P*-values were calculated by Kruskal-Wallis’s test and Dunn’s correction. NS (not significant), *P*-value >0.05; *, *P*-value <0.05; **, *P*-value <0.01; ***, *P*-value <0.001; ****, *P*-value <0.0001.

To further analyze the morphology of ∆*sltA*, additional germination and growth experiments were performed. Conidia were tracked over a period of 16 h, cell area and loss of circularity (a proxy for germination) were measured, and the data of the first 8 h were used to analyze germination kinetics (see Supplementary files 1 at https://figshare.com/s/a7900b7d20abc2b421a9). ∆*sltA* conidia started to break dormancy before WT conidia and those from a reconstituted ∆*sltA* isolate (sltArec) as observed by a significant difference in conidia size after 3 h (Fig. 2A). After 4 h, ∆*sltA* conidia were 37% and 34% larger compared to the WT and sltArec strains (Dunn’s test *P*-value <0.0001 and <0.0001, respectively) (Table 2). ∆*sltA* conidial circularity dropped significantly faster compared with the WT and sltArec strains representing rapid germ tube emergence (Fig. 2B), which was also shown by the percentage germinated conidia, after 5 h, 6 h, and 7 h (Table 2). Fifteen percent (95% CI 8.01–27.52) of ∆*sltA* conidia germinated after 5 h compared with 3% (95% CI 0.73–9.21), and 1% (95% CI 0.16–5.10) in WT (*P*-value <0.05) and sltArec (*P*-value <0.001) conidia, respectively. After 6 h, the percentage-germinated ∆*sltA* conidia increased to 83% (95% CI 70.26–90.62) compared with 33% (95% CI 23.70–44.58) and 30% (95% CI 22.05–39.15) in WT (*P*-value <0.0001) and sltArec (*P*-value <0.0001) conidia, respectively. To assess if the conidia of each strain swelled similarly in terms of area increase, we calculated the percentage of swelling per conidium before the germ tube emerged. All strains showed a similar area increase before switching to polarized growth (Fig. 2C). The rapid gemination phenotype in the ∆*sltA* strain suggests a negative regulatory role during germination for SltA.

**TABLE 2:** Conidial area and germination rate of the WT, ∆*sltA*, and sltArec strains^*a*^

Strain	Mean area^2^ 0 h in μm (SD)	Mean area^2^ 4 h in μm (SD)	Mean increase after 4 h of development in % (SD)	Germination rate 5 h in % (95% CI)	Germination rate 6 h in % (95% CI)	Germination rate 7 h in % (95% CI)	Germination rate 8 h in % (95% CI)
WT	13.86 (1.27)	18.29 (3.17)	132 (21.90)	3 (0.73, 9.21)	33 (23.70, 44.58)	85 (75.62, 91.61)	89 (80.34, 94.50)
∆*sltA*	13.07 (1.42)	22.08 (3.73)	169 (21.43)	15 (8.01, 27.52)	83 (70.26, 90.62)	98 (89.88, 99.66)	100 (93.12, 100.00)
sltArec	13.57 (1.31)	18.27 (3.06)	135 (20.32)	1 (0.16, 5.10)	30 (22.05, 39.15)	85 (77.08, 90.58)	97 (92.08, 99.04)

^*a*^ CI, confidence Interval; SD, standard deviation of the mean.

Using time-lapse live cell imaging, we analyzed the hyphal elongation phenotype of the ∆*sltA* strain (see Supplementary files 2 at https://figshare.com/s/59bae2cf7e50b357cc5e). In ∆*sltA,* hyphal tips displayed a hyper-branching phenotype with blunted tips (indicated by the arrows in Fig. 1B) when compared with WT and sltArec (Fig. 1C; Fig. 2E). In the WT and sltArec strains, new branches emerged at a mean distance of 81.52 µm and 74.17 µm, respectively, from the apical tip which generated a new lateral branch (Fig. 2D). In the ∆*sltA* strain, new branches emerged at a mean distance of 21.30 µm from the apical tip, which was the result of tip splitting events observed in ∆*sltA* (Fig. 2D). Splitting of the apical tip into two independently functioning axes is defined as dichotomous branching. In ∆*sltA*, tip splitting was observed in the majority of branch events and this was quantified by measuring the distance from the apical tip to the position of the newly emerging branch (56% <7.5 μm, Fig. 1D; Fig. 2D). Fig. 2D shows that the distance in the majority of branch events was close to zero in ∆*sltA*, representing tip splitting. This was not observed in WT and sltArec strains (0% and 1% <7.5 μm, respectively), which suggests a role for SltA in maintaining hyphal polarity and normal hyphal elongation. Hyphae of the ∆*sltA* strain were wider than their WT and sltArec hyphae counterparts (Fig. 2F); however, this was also associated with a reduced hyphal elongation rate (Fig. 2G). These results show that SltA is required for normal conidial germination and hyphal development.

### Transcriptomic analysis of germination and EHG

#### Exploratory and differential expression analysis

To assess the role of SltA in the regulation of gene expression during germination and EHG, a time-series transcriptomic analysis was carried out. RNA was extracted from dormant conidia (i.e., 0 h) and during isotropic growth (i.e., 2 h and 4 h), polarized growth (i.e., 6 h and 8 h), and EHG (i.e., 12 h and 16 h). Principal component (PC) analysis performed on the transcriptomic data revealed that the largest variance was caused by time with a large change from dormant state to isotropically swelling, and from polarized growth to EHG (Fig. S1). The largest variation was observed between the different growth phases (PC1 51.79% variance); variation between the strains was very modest and only observed when PC1 and PC6 (1.51% variance) were plotted (Fig. S1).

**Fig 3:**
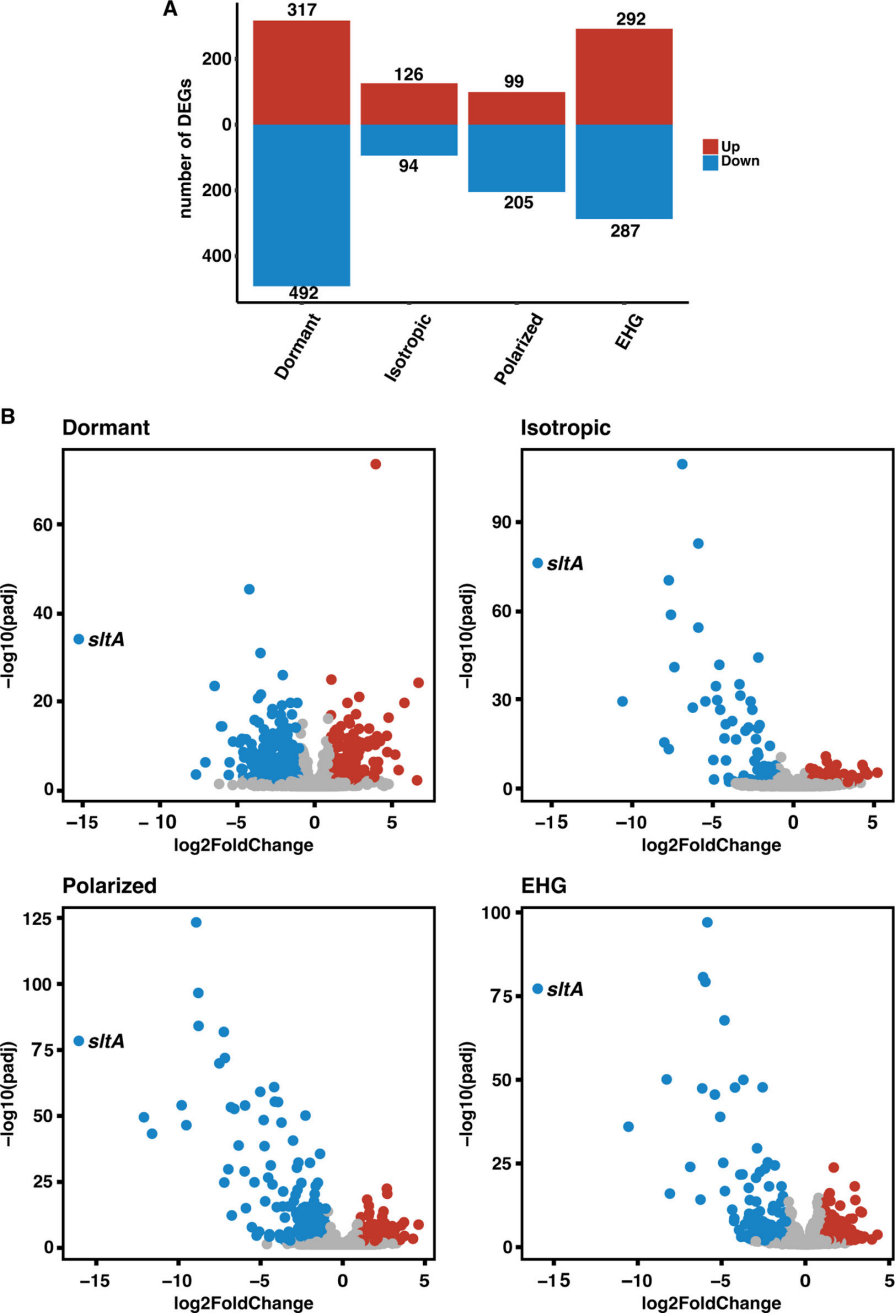
Differentially expressed genes (DEGs) during each growth phase when ∆*sltA* was compared with the WT strain. (**A**) Number of identified DEGs during each growth phase (i.e., dormant phase, isotropic swelling phase, polarized growth phase, and early hyphal growth (EHG) phase). Volcano plots for each growth phase showing the log2 fold change (Log2FC) and adjusted *P*-adjusted values. *sltA* is labelled in the plots, and showed a downregulation of <–15.00 (Log2FC).

The transcriptome of ∆*sltA* was compared with the transcriptome of the WT strain during each growth phase (i.e., dormant, isotropic growth, polarized growth, and EHG), and DEGs were identified [Log2FC ≥1 or ≤–1; adjusted *P*-value ≤0.05 (for the full overview of ∆*sltA* vs WT results, see Tables S2–S5)]. Each of the growth phases is heterogenous but were identified based on the majority of cells in that phase. The ∆*sltA* strain showed rapid germination, and therefore had more polarized cells after 6 h and 8 h compared with the WT strain. Since most of the cells in the WT strain were also polarized after 6 h and 8 h, we compared each WT growth phase with the same ∆*sltA* growth phase. Additionally, this clustering was also observed in the PC analysis (Fig. S1). In dormant conidia, 317 genes were upregulated and 492 genes were downregulated. During isotropic growth, polarized growth, and EHG, 126, 99, and 292 genes were upregulated, and 94, 205, and 287 genes were downregulated, respectively (Fig. 3A). Volcano plots of each growth phase showed a much larger distribution in downregulated genes compared with the upregulated genes as Log2FC values range from –1.00 to –12.10 and 1.00 to 6.73, respectively (Fig. 3B). This pattern was clearly visible during isotropic, polarized, and EHG.

**Fig 4:**
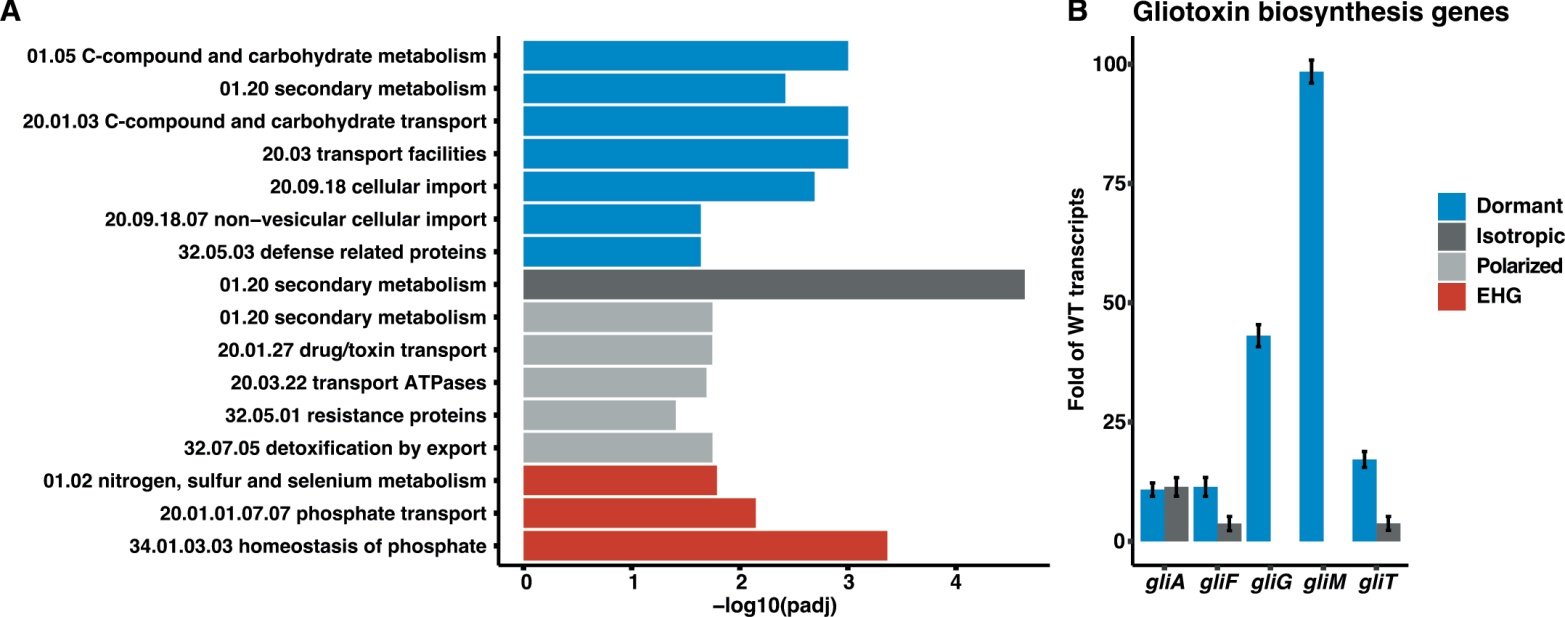
Functional category (FunCat) enrichment analysis and gliotoxin expression during each growth phase. (**A**) FunCat analysis using the DEGs identified during each of the growth phases (i.e., dormant phase, isotropic swelling phase, polarized growth phase, and early hyphal growth (EHG) phase). (**B**) Expression levels of gliotoxin biosynthesis genes enriched during the dormant phase and isotropic swelling. Expression values are shown as fold change of the WT transcript levels. Error bars represent the SE of the fold change on each growth phase.

To assess if the subsets of genes of each growth phase were enriched for specific processes, a functional enrichment analysis was performed on the DEGs of each growth phase. Despite the fact that many functional categories for filamentous fungi are poorly defined, we were able to identify enriched categories for each growth phase using FunCat classification ontology (Table S6; Fig. 4A). Enriched categories in dormant ∆*sltA* conidia were C-compound and carbohydrate metabolism, secondary metabolism, C-compound and carbohydrate transport, transport facilities, cellular import, non-vesicular cellular import, and defense-related proteins. Enriched categories in isotropically growing conidia were secondary metabolism and, during polarized growth, were secondary metabolism, drug/toxin transport, transport ATPases, resistance proteins, and detoxification by export. Enriched categories during EHG were nitrogen, sulfur and selenium metabolism, phosphate transport, and homeostasis of phosphate.

The classification ontology showed secondary metabolism was enriched in dormant conidia (99 genes), swelling conidia (42 genes), and during polarized growth (37 genes) in ∆*sltA*. To further analyze if specific secondary metabolite gene clusters were enriched, we performed a detailed analysis of genes within previously reported biosynthetic gene clusters (BGC) (40) (Table S6). Components of the gliotoxin (GT) BGC were enriched in dormant and swelling conidia [*gliA* (AFUB_075760)*, gliF* (AFUB_075780), and *gliT* (AFUB_075790)] and dormant conidia [*gliM* (AFUB_075730) and *gliG* (AFUB_075740)]. In dormant conidia, levels of *gliM*, *gliG*, *gliA*, *gliF*, and *gliT* transcripts were vastly higher (10.84- to 98.43-fold) in the ∆*sltA* strain compared with the WT strain (Fig. 4B). In isotropically growing conidia, levels of *gliA*, *gliF*, and *gliT* increased but to a lesser extent (3.72- to 11.41-fold) in the ∆*sltA* strain compared with the WT strain (Fig. 4B). However, the relevance of GT production during germination is currently unclear.

#### Analysis of genes associated with germination initiation

Initiation of germination requires sensing of external signals via signal transduction pathways. A carbon source-sensing pathway is involved in early events of germination and consists of a heterotrimeric G protein and cAMP-PKA signaling pathway (41). As ∆*sltA* conidia start to swell earlier than the WT strain, the expression levels of the genes involved in the signaling pathways associated with the onset of germination were assessed. The alpha subunit *gpaB* (AFUB_012410) of the heterotrimeric G protein was upregulated in the ∆*sltA* strain together with the catalytic subunit *pkaC* (AFUB_027890) of the cAMP-PKA signaling pathway (Table 3). Other signaling pathways associated with germination were also assessed such as the Ras signaling pathway and the calcineurin pathway. However, no genes involved in these pathways were found to be differentially expressed in the ∆*sltA* strain compared with the WT strain.

**TABLE 3:** Differentially expressed genes (∆*sltA* vs WT)

Gene ID	Name	Description^*b*^	Log2FC^*a*^
Signal transduction			
Dormant			
AFUB_012410	*gpaB*	G protein complex alpha subunit GpaB	1.01
AFUB_027890	*pkaC*	cAMP-dependent protein kinase catalytic subunit PkaC1	1.14
Cell wall biosynthesis			
Dormant			
AFUB_015530	*aspf9*	Extracellular cell wall glucanase Crf1/allergen Asp F9	–1.31
AFUB_029980	*eng2*	GPI-anchored endo-beta-1,3-glucanase, putative	–1.1
AFUB_041890		Putative family 18 chitinase	–1.15
AFUB_047490	*ags1*	Alpha-1,3-glucan synthase, putative	–2.34
AFUB_050810	*eng4*	Concanavalin A-like lectin/glucanase superfamily protein, putative	–2.52
AFUB_061710	*eng8*	Concanavalin A-like lectin/glucanase superfamily protein, putative	–1.17
AFUB_073300		Methionine aminopeptidase type I, putative	–1.07
AFUB_078380	*scw4*	Putative cell wall glucanase	–1.4
AFUB_078400	*fks1*	1,3-beta-glucan synthase catalytic subunit	–1.46
AFUB_078410	*gel7*	1,3-beta-glucanosyltransferase, putative	–4.09
AFUB_095040	*ap1*	Aspartic endopeptidase, putative	–1.13
AFUB_000280	*bglH*	Beta-glucosidase, putative	1.30
AFUB_005640	*eng3*	Concanavalin A-like lectin/glucanase superfamily protein, putative	2.44
AFUB_021050		Alpha-1,3-glucanase/mutanase, putative	2.90
AFUB_032990		Extracelular serine carboxypeptidase, putative	1.30
AFUB_085200	*chiB1*	Class V chitinase	1.44
AFUB_099700	*exg10*	Pectate lyase superfamily protein	1.28
Isotropic			
AFUB_052460	*chi100*	Class V chitinase	1.59
AFUB_078410	*gel7*	1,3-beta-glucanosyltransferase, putative	1.45
AFUB_086160	*metAP*	Methionine aminopeptidase, type II, putative	1.17
AFUB_091720	*bglL*	Putative secreted 1,4-beta-D-glucan glucanhydrolase	1.62
Polarized			
AFUB_095070	*crh1*	Putative cell wall glucanase	–4.72
AFUB_097210	*cpdS*	Serine carboxypeptidase, putative	–2.01
AFUB_029980	*eng2*	GPI-anchored endo-beta-1,3-glucanase, putative	1.11
AFUB_086210		NlpC/P60-like cell-wall peptidase, putative	2.85
EHG			
AFUB_004590	*engl1*	Endo-1,3(4)-beta-glucanase	–1.19
AFUB_083440	*s1*	Carboxypeptidase, putative	–1.28
AFUB_086160	*metAP*	Methionine aminopeptidase, type II, putative	–1.34
AFUB_095070	*crh1*	Putative cell wall glucanase	–2.75
AFUB_097210	*cpdS*	Serine carboxypeptidase, putative	–1.87
AFUB_024920	*dppV*	Secreted dipeptidyl peptidase	1.21
AFUB_049670	*cp6*	Carboxypeptidase S1, putative	2.09
AFUB_074600	*oct1*	Metallopeptidase	1.09

^*a*^ Log2FC, log2 fold change.

^*b*^ GPI, Glycosylphosphatidylinositol.

#### Analysis of genes associated with cell wall biosynthesis and modifications

Cell wall biosynthesis and modifications are important for the plasticity of the cell wall during isotropic swelling and outgrowth of the germ tube, followed by tip elongation and branching (42, 43). The ∆*sltA* mutant showed rapid germ tube emergence and hyphal elongation and branching defects; therefore, the expression of genes associated with cell wall biosynthesis and modifications was assessed.

**Fig 5:**
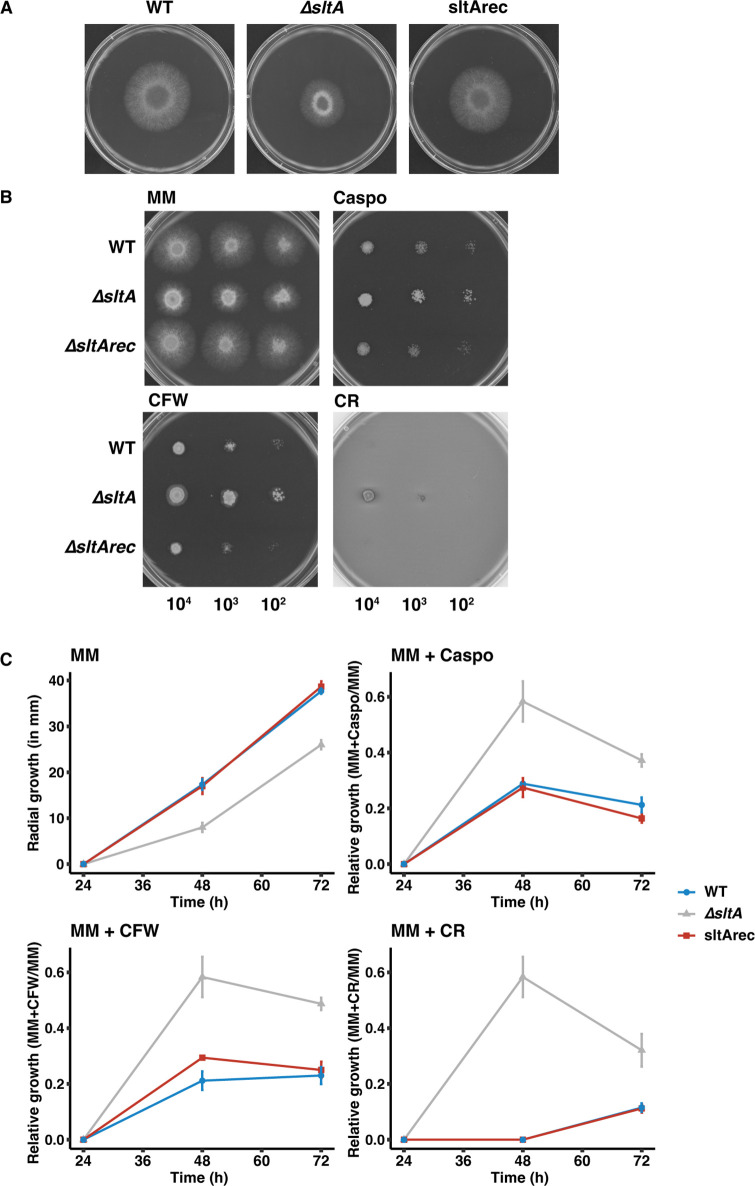
Colonial growth phenotypes and cell wall stress of the WT, ∆*sltA*, sltArec strains. (**A**) Colonial growth phenotypes of each strain grown on solid minimal medium (MM). Susceptibility of the ∆*sltA* strain was analyzed by (**B**) serial 10-fold dilutions and (**C**) relative radial growth on MM supplemented with caspofungin (caspo), calcofluor white (CFW), and Congo red (CR). The error bars represent SE of the mean of three independent experiments.

DEGs associated with cell wall biosynthesis and modifications were identified in dormant conidia, swelling conidia, and during polarized and EHG in the ∆*sltA* strain (Table 3). Among the DEGs were glucanases, chitinases, a glucanosyltransferase, glucan synthases, and several peptidases. Cell wall glucanase *crh1* mRNA levels highly decreased during polarized and EHG in the ∆*sltA* strain compared with the WT strain, –26.38- and –6.74-fold, respectively. As several genes involved in the synthesis and reorganization of glucan and chitin were differentially expressed, we exposed the WT, ∆*sltA*, and sltArec strains to cell wall inhibitors such as caspo, CFW, and CR. The ∆*sltA* strain showed reduced radial growth on MM compared to the WT and sltArec strains (Fig. 5A). With cell wall formation inhibitors, the ∆*sltA* strain showed no marked increased susceptibility grown in the presence of the cell wall stressors compared to the WT and sltArec strains (Fig. 5B). When the radial growth was normalized to radial growth in control conditions (MM), hyphae showed even better growth (Fig. 5C).

#### Analysis of genes associated with the Spk and apical vesicle transport

The ∆*sltA* strain showed a hyper-branching and tip splitting phenotype when grown in RPMI-1640 medium. This phenotype may be the result of dysregulation of genes associated with the Spk and hyphal elongation. However, no genes associated with the polarisome, Arp2/3 complex, exocyst, secretion guanosine triphosphate hydrolase enzymes and interacting proteins, soluble N-ethylmale-imide-sensitive factor-attachment protein receptors in vesicular transport, endocytosis, and exocytosis were differentially expressed in the ∆*sltA* strain during germination and EHG. This suggests that vesicle fusion with endosomes and the plasma membrane is not affected in the ∆*sltA* mutant.

### Specific genes in the regulon of SltA are required for appropriate germination

**Fig 6:**
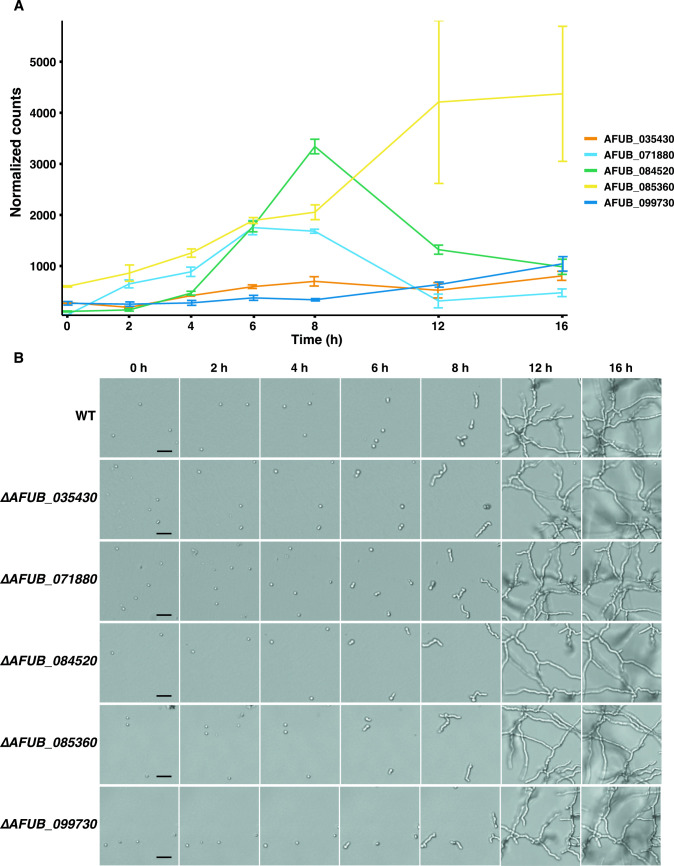
Expression values in the WT strain of the selected genes and phenotypic characterization of the knockout strains. (**A**) Normalized counts of five selected genes in the WT strain during dormant, isotropic, polarized, and EHG phase. (**B**) Germination and early hyphal growth phenotype of the five knockout strains, size bar represents 25 µm.

To identify SltA target genes that might be responsible for the early germination and distorted EHG phenotype observed in ∆*sltA*, the most highly downregulated genes during all analyzed growth phases were assessed (i.e., ∆*sltA* vs WT for each growth phase). The average of all Log2FC values per gene were calculated and sorted from smallest to largest; genes were only included when significance thresholds were met for all analyzed growth phases. The top 12 genes with the lowest average expression values are shown in Table 4 . All of the genes showed Log2FC values of –2.07 to –12.10 during the isotropic, polarized, and EHG phases. The regulation of five of these genes was particularly notable as their expression levels in the WT strain increased during germination (Fig. 6A). Previously, Du et al. (44) showed SltA regulates the expression of ergosterol biosynthesis and drug efflux-related genes by directly binding to the conserved 5′-AGGCA-3′ motif in their promoter regions. The SltA binding motif was present multiple times in the promoter region of the five genes, indicating direct regulation by SltA (Fig. S3). Transcript levels of the genes AFUB_035430 and AFUB_099730 gradually increased during germination and EHG. Transcript levels of AFUB_071880 and AFUB_084520 increased during polarized growth, then, during EHG, decreased again. Transcript levels of AFUB_085360 increased during germination and EHG. Hence, their role in the early growth phases was assessed further.

**TABLE 4:** Highly downregulated genes (∆*sltA* vs WT)

GeneID	Name	Description	Log2FC^*a*^			
			Dormant	Isotropic	Polarized	EHG
AFUB_084520		Nucleotide-sugar transporter	–6.05	–10.60	–12.10	–8.27
AFUB_084540		Glycosyl transferase, putative	–7.06	–5.46	–11.61	–8.08
AFUB_084560		Conserved hypothetical protein	–4.43	–4.81	–8.79	–10.55
AFUB_071880	*fedD*	Low-affinity ferrous iron transport protein	–2.84	–6.90	–8.92	–6.16
AFUB_101750	*bsc1*	Mitochondrial chaperone ATPase, putative	–5.98	–8.01	–6.34	–4.25
AFUB_085360		Hypothetical protein	–2.47	–7.73	–8.78	–5.40
AFUB_034540	*plb3*	Lysophospholipase B	–1.64	–5.89	–9.82	–5.96
AFUB_084550		N-acetylglucosaminyl-phosphatidylinositol deacetylase, putative	–4.03	–4.54	–7.52	–6.25
AFUB_049120		Hypothetical protein	–2.75	–7.73	–7.22	–3.23
AFUB_058080		Amidase	–2.56	–4.18	–6.96	–6.86
AFUB_099730		Hypothetical protein	–1.37	–5.89	–7.17	–5.84
AFUB_046500		Hypothetical protein	–2.80	–7.59	–6.81	–2.07

^*a*^ EHG, early hyphal growth; Log2FC, log2 fold change

**Fig 7:**
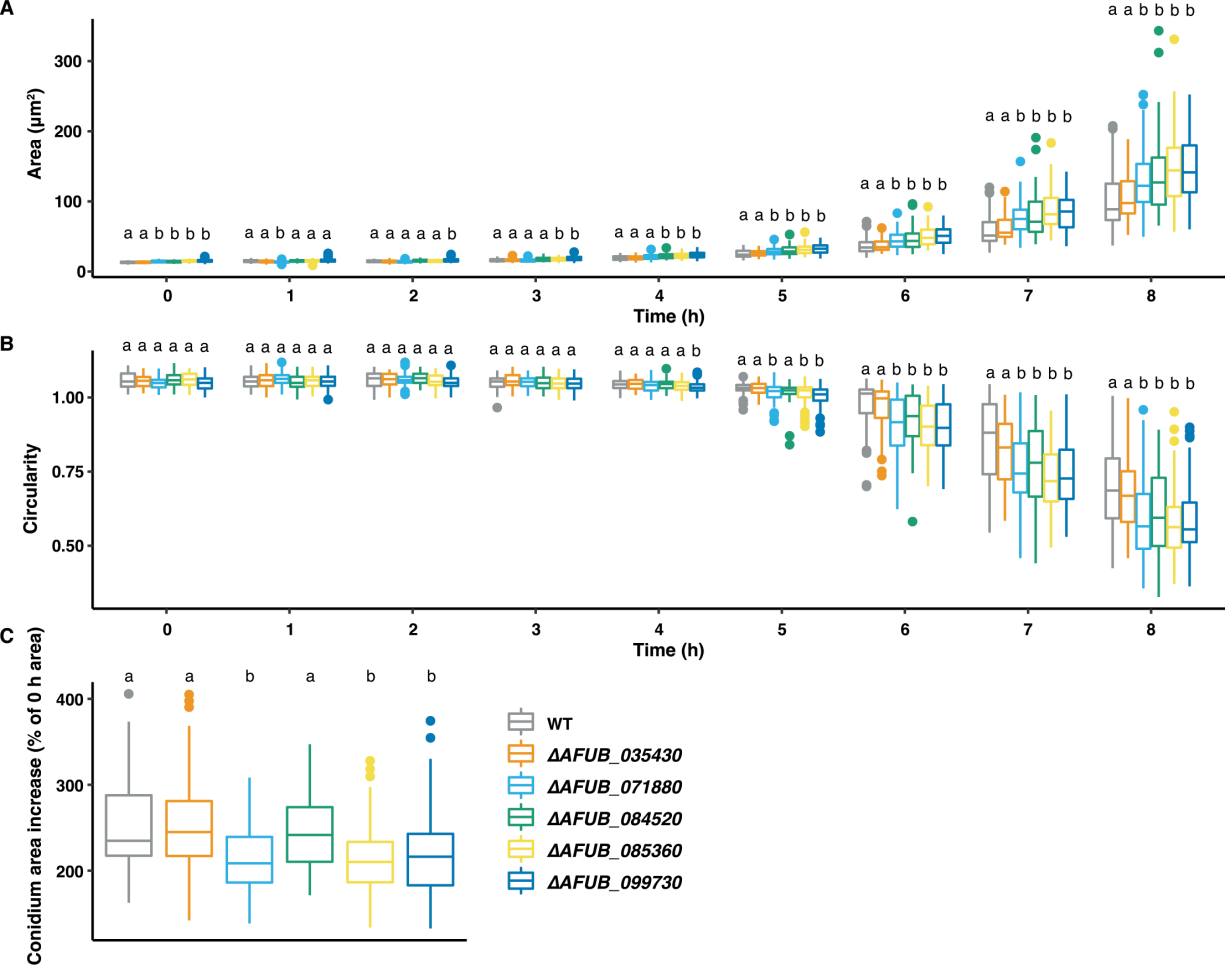
Strain ∆*AFUB_071880*, ∆*AFUB_084520*, and ∆*AFUB_099730* are required for appropriate germination. (**A**) Fungal area of the WT, ∆*AFUB_035430*, ∆*AFUB_071880*, ∆*AFUB_085360*, ∆*AFUB_084520*, and ∆*AFUB_099730* strains were measured during 8 h of germination at 37 °C. (**B**) Fungal object circularity of the WT, ∆*AFUB_035430*, ∆*AFUB_071880*, ∆*AFUB_085360*, ∆*AFUB_084520*, and ∆*AFUB_099730* strains were measured during 8 h of germination. Round conidia have a circularity of >1.00; this number decreases when cells switch to polarized growth. (**C**) Conidium area increase in percentage compared with the 0 h area, i.e., conidial area before germ tube emergence was compared with the conidial area at 0 h. *P*-values were calculated by Kruskal-Wallis’s test and Dunn’s correction; all strains were compared with the WT strain. Values marked with different letters are significantly different from each other (*P*<0.01).

The effect of loss of these five genes on the germination was analyzed by tracking conidia for the first 16 h of growth (Fig. 6B). No apparent hyphal branching and elongation defects were observed during EHG. Cell area and circularity were measured, and the data of the first 8 h was used to analyze germination kinetics (see Supplementary files 1 at https://figshare.com/s/a7900b7d20abc2b421a9). We compared the ∆*AFUB_035430*, ∆*AFUB_071880*, ∆*AFUB_085360*, ∆*AFUB_084520*, and ∆*AFUB_099730* strains with the WT strain for area increase and drop of circularity during 8 h of growth (Fig. 7A and B). Dormant conidia of all null strains, except for ∆*AFUB_035430*, were slightly larger compared with the WT strain (Table 5; Fig. 7A). This was also observed after 4 h of growth. However, the conidial area increase during the first 4 h of growth was similar between all analyzed strains (Table 5). Consistent with the phenotype exhibited by the *sltA*, null mutant conidial circularity of the ∆*AFUB_099730* strain dropped significantly faster compared with the WT strain, representing rapid germ tube emergence (Fig. 7B). Similar results were observed after 5 h when strains ∆*AFUB_071880* and ∆*AFUB_084520* were compared with the WT strain. This was also shown by the percentage of germinated conidia (Table 5), which was 10% in ∆*AFUB_071880* (95% CI 5.15–18.51, *P*-value >0.05), 10% in ∆*AFUB_084520* (95% CI 5.15–18.51, *P*-value >0.05), and 20% in ∆*AFUB_099730* (95% CI 13.74–29.16, *P*-value <0.01) compared with 4% in the WT strain (95% CI 1.28–10.45). After 6 h, percentage of germinated conidia in strains ∆*AFUB_071880*, ∆*AFUB_085360*, ∆*AFUB_084520*, and ∆*AFUB_099730* increased to 71% (95% CI 60.53–80.01, *P*-value <0.0001), 57% (95% CI 43.27–69.97, *P*-value <0.05), 76% (95% CI 65.86–84.24, *P*-value <0.0001), and 75% (95% CI 65.58–82.15, *P*-value <0.0001), respectively, compared with 33.75% (95% CI 24.35–44.64) in the WT strain (Table 5). As a rapid germination phenotype was observed in strains ∆*AFUB_071880*, ∆*AFUB_084520*, and ∆*AFUB_099730*, but no difference in swelling of the conidia, we calculated the percentage of swelling per conidium before the germ tube emerged. ∆*AFUB_071880*, ∆*AFUB_084520*, and ∆*AFUB_099730* conidia increased their conidial area less before they switched to polarized growth and the germ tube emerged (Fig. 7C).

**TABLE 5:** Conidial area and germination rate of the WT, ∆*AFUB_035430*, ∆*AFUB_071880*, ∆*AFUB_085360*, ∆*AFUB_084520*, and ∆*AFUB_099730* strains

Strain	Mean area^2^ 0 h in μm (SD)	Mean area^2^ 4 h in μm (SD)	Mean increase after 4 h of growth in % (SD)	Germination rate 5 h in % (95% CI)	Germination rate 6 h in % (95% CI)	Germination rate 7 h in % (95% CI)	Germination rate 8 h in % (95% CI)
WT	13.23 (1.31)	19.20 (3.22)	146 (24.19)	4 (1.28, 10.45)	34 (24.35, 44.64)	75 (64.52, 83.19)	98 (91.33, 99.31)
∆*AFUB_035430*	13.13 (1.35)	19.65 (3.36)	150 (23.27)	1 (0.25, 7.66)	40 (29.33, 51.70)	86 (75.66, 92.05)	99 (92.34, 99.74)
∆*AFUB_071880*	14.27 (1.76)	20.54 (3.71)	144 (24.77)	10 (5.15, 18.51)	71 (60.53, 80.01)	98 (91.33, 99.31)	100 (95.42, 100.00)
∆*AFUB_085360*	14.18 (1.56)	22.04 (4.43)	155 (25.30)	4 (1.13, 13.71)	57 (43.27, 69.97)	94 (83.48, 97.90)	100 (92.73, 100.00)
∆*AFUB_084520*	15.03 (1.63)	22.34 (3.80)	149 (20.00)	10 (5.15, 18.51)	76 (65.86, 84.24)	100.00 (95.42, 100.00)	100 (95.42, 100.00)
∆*AFUB_099730*	15.19 (2.02)	23.79 (4.58)	157 (25.04)	20 (13.74, 29.16)	75 (65.58, 82.15)	97 (91.78, 99.00)	100 (96.40, 100.00)

Sequence analysis of the five selected genes showed that gene AFUB_035430 encodes a protein of 387 amino acids containing an alpha/beta hydrolase domain. Orthologous genes are only found in filamentous Eurotiales and Hypocreales species, mainly *Trichoderma* and *Aspergillus* (Fig. S2), and not yet characterized. The gene AFUB_071880 encodes a 541 amino acid transmembrane transporter containing a low-affinity iron permease sequence similar to *Saccharomyces cerevisiae* FET4. In *S. cerevisiae* FET4 encodes a low iron transporter also involved in zinc, copper, cobalt, manganese, and cadmium transport (45 - 49). Orthologous genes are found in filamentous fungi from the orders Pleosporales, Cheatothyriales, and Eurotiales (Fig. S2). The gene AFUB_085360 encodes the fungal sterol-specific aminoacyl-tRNA transferase (ATT) ergosterol-3β-O-glycine synthase ErgS (50). This enzyme only uses Gly-tRNA^gly^ to produce an independent glycyl-tRNA synthetase to transfer glycine onto the 3β-OH of ergosterol. This enzyme was only found in Ascomycota and, together with ergosteryl-3β-O-L-aspartate synthase ErdS, they seem to constitute a subfamily of lipid-modifying ATTs (51). The gene AFUB_084520 encodes a 354 amino acid membrane nucleotide-sugar transporter predicted to transport nucleotide sugars from the cytoplasm into golgi vesicles (52). The gene also has a multidrug-resistant efflux transporter similar to EmrE from *Escherichia coli*, and also seems unique to the genus *Aspergillus* as orthologous are only found in *A. fumigatus*, *A. clavatus*, and *A. fischeri* (Fig. S2). The gene AFUB_099730 encodes a 78 amino acid protein containing predicted transmembrane domains expected to be outside the membrane in the extracellular region. The gene seems to be unique to the genus *Aspergillus* as only orthologous are found in *A. fumigatus*, *A. clavatus*, and *A. fischeri* (Fig. S2).

## DISCUSSION

In this study, we followed up on a previous screening of 484 TFs for germination by analyzing 13 TF null strains for phenotypic changes during germination phases (i.e., dormant, isotropic swelling, and polarized growth) and EHG. One of the null strains (∆*sltA*) showed an early onset of isotropic growth and slower growth of fatter germ tubes and early hyphae. Further hyper-branching, meandering growth, and tip splitting were observed. We explored, in detail, the germination and EHG of ∆*sltA* and the transcriptomic situation during these phases. Previous studies have demonstrated that the C_2_H_2_ zinc finger TF SltA functions as a regulator for cation homeostasis (53, 54), is important for azole resistance by regulating the ergosterol biosynthesis pathway (44), and plays a role in secondary metabolite production and virulence (16). In our study, we demonstrated a novel role for TF SltA in germination and EHG by phenotypic analysis.

The loss of SltA resulted in an early germination phenotype such as rapid swelling of conidia and rapid germ tube emergence when compared to the WT strain. ∆*sltA* conidia showed similar increase of size during isotropic growth as the WT and sltArec conidia, but started to swell at an earlier time point and, subsequently, the germ tube emerged earlier. After germ tube emergence, elongation of the hyphae also showed abnormal growth such as hyper-branching, curly and wider hyphae, and splitting of the apical tip. The elongation rate of the ∆*sltA* hyphae is low, but the broader hyphae suggest a similar amount of cell expansion. This could also indicate that functioning of the Spk, as vesicle supply center is less apical and vesicles fuse at higher levels at more subapical membranes.

The gene set enrichment analysis showed secondary metabolism significantly enriched during all growth phases except for EHG. The secondary metabolite GT gene cluster was highly upregulated in dormant and isotropically swelling conidia. GT is a virulence factor which plays a role in the pathobiology as it modulates the immune response and induces apoptosis in different cell types (55). The GT biosynthesis genes *gliA*, *gliF*, *gliT*, *gliM*, and *gliG* were upregulated in the ∆*sltA* strain. The *gliA*, *gliT*, and *gliM* genes were also upregulated in *A. fumigatus* ∆*mbsA* and ∆*rgdA* strains which resulted in a fivefold higher production of GT in both strains (56, 57). Virulence, however, of the ∆*sltA*, ∆*mbsA*, and ∆*rgdA* strains were reduced in neutropenic mice when compared to the WT strain (16, 56, 57). Despite the upregulation of GT biosynthesis genes and supposedly elevated GT levels, the ∆*sltA* strain showed reduced virulence, which may be associated with the hyphal polarity defects. The relevance of GT production in conidia and during germination is unclear; however, accumulation of mycotoxins in spores has been observed before. The mycotoxin trypacidin in *A. fumigatus* affects the phagocytic interaction between conidia and amoebae, suggesting trypacidin is responsible for predation avoidance (58). Additionally, amoebae and macrophages were more sensitive to trypacidin, which suggests it could have a protective function in the environment and during infection. GT was also found to have an amoebicidal effect, and may make conidia less attractive for amoebae in the environment. The elevated transcript levels of GT biosynthesis genes in ∆*sltA*, however, remain an enigma.

Cell wall remodeling and cell wall biosynthesis are associated with conidial swelling and polarized growth in *Aspergillus* spp (10). Several glucanases, glucan synthases, and chitinases were differentially expressed during these growth phases in the ∆*sltA* strain. The ∆*sltA* strain showed similar tolerance to CR, caspo, and CFW when compared to the WT strain. Liu H et al. analyzed cell wall stress resistance of the ∆*sltA* strain and found near wild-type susceptibility for the tested stressors (i.e., caspo, CFW, and CR) (16). In another study, Liu Z et al. also analyzed CR and CFW susceptibility in ∆*sltA*, and found a resistant phenotype compared to the WT strain (59). However, when caspo was tested, the ∆*sltA* strain was more sensitive compared to the WT strain. This interesting difference could potentially be due to strain differences; however, a side-by-side comparison has not been performed yet. The cell wall stressors bind to chitin and/or β-glucans (60); therefore, the non-WT susceptibility in ∆*sltA* found in this study suggests that the cell wall architecture may be different compared to the WT strain, which could affect conidial swelling and polarized growth.

The cell wall biosynthesis and remodeling enzymes are transported to the apical tip by a continuous flow of secretory vesicles. First, the vesicles accumulate in the Spk before moving further to the tip membrane where they fuse (8). However, no genes involved in these processes showed differential expression in the ∆*sltA* strain. The place of vesicle fusion and, therefore, the direction of growth are orchestrated by the position of the Spk, cell end markers, and other proteins involved in the process of exocytosis (8). Displacement of the Spk can cause serious changes in hyphal development and morphology. Dislocation of the Spk in hyphae of *Rhizoctonia solani* resulted in a decline in elongation rate, a rounded apex, and increased diameter (13). Similar characteristics of hyphal development were observed in the ∆*sltA* strain, which indicates that positioning and tethering of the Spk is less apical, and vesicles fuse at more subapical membranes. This could be related to actin fibers or actin-related genes; however, this was not detected by the enrichment analysis. In *Geotrichum candidum*, subapical swelling is observed with actin disruption causing abnormal expansion and delocalizing exocytosis (61). Tip splitting, meandering growth, and wider hyphae could very well be caused by a less stable vesicle supple center. A Spk that disappears and reappears results in a bulging growth and widening hyphae (62), as observed in hyphae of the ∆*sltA* strain. In *R. solani*, dislocation of the Spk declined the elongation speed abruptly, and the apex became rounded and increased in diameter (13). In a temperature-sensitive apically branching mutant of *A. niger*, the original Spk disappeared and two new Spk producing localized zones of wall expansion with deformations of the hypha appeared as a consequence (63). Taken together, these observations suggest that the stability and positioning of the Spk could be affected in this mutant.

No Spk-associated genes were found to be differentially expressed in the ∆*sltA* strain; however, cell polarity and hyphal elongation require orchestrated regulation of signaling, cytoskeletal elements, and membrane trafficking and delivery of vesicles. The hyphal defects observed in ∆*sltA* may be an indirect effect of these processes being perturbed and could, together with the differentially expressed cell wall and ergosterol-associated genes, result in altered hyphal tip growth. This may in the same way affect germination. More research is necessary to identify the relation between loss of SltA and the disturbed processes leading to hyphal elongation defects and rapid germination.

To assess which genes may be involved in the rapid germination and unstable hyphal elongation phenotype, we selected highly downregulated genes and observed the first 16 h of development of five knockout strains. No difference in hyphal development was observed, but three of the selected strains (∆*AFUB_071880*, ∆*AFUB_084520*, and ∆*AFUB_099730*) showed rapid germ tube emergence compared with the WT strain. The conidia, however, did not start to swell at an earlier time point, but increased less in terms of conidial area before the germ tube emerged. Interestingly, the genes AFUB_084520 and AFUB_099730 are unique to the genus *Aspergillus* and not present in all species. Both genes are coding for transmembrane proteins, AFUB_084520 encodes a nucleotide sugar transporter, and AFUB_099730 is a very small (78 amino acids) protein containing transmembrane domains. Further research is needed to unravel how these transmembrane proteins play a role in rapid emergence of the germ tube. Taken together, we have identified two different mechanisms for rapid germination in *A. fumigatus* conidia: conidia started to swell earlier and subsequently switched to polarized growth faster, and conidia swelled at a similar rate as seen in the WT strain, but switched to polarized growth faster.

In summary, we have screened 13 TF null mutants for their importance during isotropic swelling, polarized growth, and EHG, and were able to identify the C_2_H_2_ zinc finger TF SltA to be involved in germination (i.e., swelling and germ tube emergence) and EHG. Phenotypic analysis showed rapid swelling of the conidia and rapid germ tube emergence. After germ tube emergence, the elongating hyphae are wider and curlier compared to the WT strain. Additionally, we observed a hyper-branching and splitting of the apical tip phenotype. A transcriptomic analysis of dormant conidia, germinating conidia, and EHG did not show a dysregulation of polar hyphal growth-related genes. The elaborate phenotypic analysis suggests that the Spk in ∆*sltA* may be unstable in terms of positioning, speed, and behavior of the Spk.

## Data Availability

Raw sequence reads are available through the NCBI Sequence Read Archive database under BioProject ID: PRJNA913092. WT strain is accessible under BioSample: 3221185 and ∆sltA strain is accessible under BioSample: SAMN32271186.

## SUPPLEMENTAL MATERIAL

TABLE S1 msphere.00076-23-s0001.xlsx Selected TFs associated with germination.

TABLE S2 msphere.00076-23-s0002.xlsx RNA-seq results for dormant phase.

TABLE S3 msphere.00076-23-s0003.xlsx RNA-seq results for isotropic phase.

TABLE S4 msphere.00076-23-s0004.xlsx RNA-seq results for polarized phase.

TABLE S5 msphere.00076-23-s0005.xlsx RNA-seq results for EHG phase.

TABLE S6 msphere.00076-23-s0006.xlsx FunCat results.

TABLE S7 msphere.00076-23-s0007.xlsx Primers and gRNAs used in this study.

Fig S1 msphere.00076-23-s0008.eps Principal component analysis of the log transformed expression data. The left plot shows PC1 on the x-axis and PC2 on the y-axis. The right plot shows PC1 on the x-axis and PC6 on the y-axis.

Fig S2 msphere.00076-23-s0009.eps Presence and absence of five selected genes in Dikarya species. To identify orthologous genes, the gene ontology information from www.fungi.ensembl.org was used (on 2022-11-18), only the high confidence orthologous genes from www.fungi.ensembl.org were used. The www.fungi.ensembl.org results were compared with the gene ontology information from www.fungidb.org.

Fig S3 msphere.00076-23-s0010.eps Promotor analysis of five selected genes. Presence of the SltA binding motif 5ʹ-AGGCA-3ʹ within the first 2,000 bp upstream of transcription start site ATG. UTR regions are highlighted when annotated.

ASM does not own the copyrights to Supplemental Material that may be linked to, or accessed through, an article. The authors have granted ASM a non-exclusive, world-wide license to publish the Supplemental Material files. Please contact the corresponding author directly for reuse.
